# Albuminuria is an independent risk factor of T4 elevation in chronic kidney disease

**DOI:** 10.1038/srep41302

**Published:** 2017-01-24

**Authors:** Xin Du, Binbin Pan, Wenwen Li, Yonghua Zou, Xi Hua, Wenjuan Huang, Xin Wan, Changchun Cao

**Affiliations:** 1 Department of Nephrology, Nanjing First Hospital, Nanjing Medical University, Nanjing, PR China; 2 Department of Nephrology, Sir Run Run Hospital, Nanjing Medical University, Nanjing, PR China; 3 Outpatient Department, Nanjing Medical University, Nanjing, PR China

## Abstract

This study was to explore the association between thyroid dysfunction and albuminuria. 581 cases with chronic kidney disease (CKD) were included in this study. The clinical characteristics consisted of sex, age, serum creatinine, urinary albumin-to-creatinine ratio (ACR), thyroid function were recorded. Estimated glomerular filtration rate (eGFR) was calculated by CKD-EPI four-level race equation. Prevalence of different thyroid diseases was calculated by chi-square test. Levels of thyroid hormone were compared among different albuminuria groups by Kruskal-Wallis test. Spearman’s correlation was used to assess the association between albuminuria and thyroid hormone. Our study showed that total T4 and free T4 were significantly different among ACR < 30 mg/g, 30–300 mg/g and >300 mg/g (P < 0.001 and =0.007, respectively). Positive correlation between T4 (total T4 and free T4) and albuminuria was evaluated by correlation analysis (P = 0.001 and <0.001, respectively). Albuminuria was an independent influence factor of T4 after adjustment for age, sex, serum creatinine, albumin, hs-CRP, smoking status, systolic blood pressure, diabetes mellitus, medication use for diabetes mellitus, eGFR, LDL-cholesterol, triglycerides, hypertension, and medication use for hypercholesterinemia. In conclusion, T4 was positively correlated with albuminuria, and it was completely not consistent with our anticipation. Further study is needed to elucidate the causation association between albuminuria and T4.

Chronic kidney disease (CKD) is a global public health problem, affecting 10–16% of the adult population worldwide^1^. CKD is characterised by low estimated glomerular filtration rate (eGFR) and high albuminuria^1^, persistent for more than three months^1^, and is associated with adverse outcomes, irrespective of hypertension^2^, diabetes^3^, age^4^, and sex^5^.

Persistent proteinuria and/or albuminuria for more than three months is a hallmark of CKD^6^. It is well established that the kidney is capable of participating in all aspects of peripheral thyroid hormone (TH) metabolism. Intact THs are filtered, reabsorbed and secreted by the kidney. A major route of iodide elimination is by urinary excretion^7^. Nephrotic syndrome is associated with changes in serum TH levels^8^. Urinary losses of binding proteins, such as thyroxine binding globulin (TBG), transthyretin or pre-albumin, albumin, and TH binded to them, result in a reduction in serum total thyroxine (TT4) and, sometimes, in total triiodothyronine (TT3) levels^9^. These hormonal changes are related both to the degree of proteinuria and to serum albumin levels^10^. Patients often remain euthyroid, because free T4 (FT4) and free T3 (FT3) levels are usually normal^10^. Thyroid is able to compensate for hormonal urinary losses keeping the patient euthyroid. However, in patients with low thyroid reserve, clinical hypothyroidism (or overt hypothyroidism) can develop^11^. Similarly, nephrotic syndrome may increase the needs of exogenous levothyroxine in patients with hypothyroidism.

It is well known that serum TT4 and sometimes FT4 were both decreased in nephrotic syndrome^7^,^8^,^9^,^10^,^11^,^12^. Serum TT4 and FT4 were negatively correlated with massive proteinuria. However, the association of albuminuria and thyroid dysfunction is unknown. Our research was to explore the associations between thyroid dysfunction and albuminuria. It is hypothesized that serum TT4 and FT4 would be also negatively correlated with albuminuria.

## Methods

### Study population

We performed a cross-sectional analysis on the database of the Laboratory Information System of the Clinical Chemistry Laboratory at Nanjing First Hospital of Nanjing Medical University to retrieve results of urinary albumin to creatinine ratio, serum creatinine, and thyroid function tests, which have been performed on 1689 inpatient adults (≥18 yr of age) consecutively referred by nephrology practitioners for routine blood testing over the last 7 yr (from August 2009 to August 2016). Different albuminuria groups were set by the level of albumin to creatinine ratio in urine (ACR) and eGFR according to the KDIGO guideline^1^. We excluded subjects who were younger than 18 years of age, women who were pregnant (given potential pregnancy-related changes in thyroid function), subjects with urinary traction infection (given potential trasient albuminuria, *N* = 15), subjects with previous thyroid disease (*N* = 3), subjects who have a history of thyroid disease or who were receiving concurrent treatment with drugs that could effect on thyroid function (lithium, amiodarone, iodine, methimazole or propylthiouracil, *N* = 1). We also excluded subjects in which thyrotropin (or thyroid-stimulating hormone, TSH), total T3, free T3, total T4 and free T4 levels were not available (*N* = 1089). Thus, this study analyzes the remaining 581 patients. Only 15 patients tested anti-thyroid antibody, 16 patients tested reverse T3, so we did not analyze them in the study. Proteinuria was performed in only 72 patients, and the association of proteinuria and thyroid dysfunction was well studied^7^,^8^,^9^,^10^,^11^,^12^, thus, we did not analyze proteinuria in the study.

The study was performed in accordance with the Declaration of Helsinki and was approved by the Institutional Review Board at Nanjing First Hospital affiliated to Nanjing Medical University (IRB no. 86-025-52271039-21). Informed consents were obtained from all participants when they were admitted to the hospital.

### Laboratory measures

Thyroid function and urinary albumin were tested by using electrochemiluminescence assay (Siemens ADVIA Centaur XP, NY, USA). The reference ranges of serum TT4, FT4, TT3, FT3 and TSH in our institute are as follows: for TSH is 0.55–4.78 mIU/L, for TT4 58.1–140.6 mmol/L, for FT4 11.5–22.7 pmol/L, for TT3 0.92–2.79 mmol/L, for FT3 3.5–6.5 pmol/L, respectively. The reference range of thyroid function was set according to the instructions from ADVIA Centaur immunoassay system^13^. Clinical hyperthyroidism is defined as lower (less than normal value) TSH with higher (more than normal value) FT4, TT4 and/or FT3, TT3; clinical hypothyroidism is defined as higher TSH with lower FT4, TT4; subclinical hyperthyroidism is defined as lower TSH with normal FT4, TT4, FT3 and TT3; subclinical hypothyroidism is defined as higher TSH with normal FT4, TT4, FT3, and TT3; euthyroid sick syndrome is defined as normal TSH with lower FT3, TT3 and/or lower FT4, TT4^14^. Enzymatic method was performed to evaluate serum creatinine (Scr) and urinary creatinine by using OLYMPUS AU5400 automatic biochemical analyzer (Olympus Corporation, Mishima, Japan). The calibrators for the enzymatic method were traceable to an isotope dilution mass spectrometric method for serum creatinine using standard reference methods NIST SRM 967^15^. All covariates including serum albumin, LDL cholesterol, triglycerides, hs-CRP, smoking status, systolic blood pressure, diabetes mellitus, medication use for diabetes mellitus, hypertension, and medication use for hypercholesterinemia were measured and recorded. Serum albumin, LDL cholesterol and triglycerides were measured by biochemical method, using OLYMPUS AU5400 automatic biochemical analyzer (Olympus Corporation, Mishima, Japan). CRP was measured by rate nephelometry.

Estimated glomerular filtration rate (eGFR) was calculated by the Chronic Kidney Disease Epidemiology Collaboration (CKD-EPI) four-level race equation^16^,^17^. The specific CKD-EPI four-level race GFR estimation equation was shown in Table 1.

Albuminuria was evaluated by ACR which was calculated as the ratio of urinary albumin to urinary creatinine. Patients were assigned into three groups according to the KDIGO guideline 2012: ^1^normal to mildly increased albuminuria (ACR < 30 mg/g); moderately increased albuminuria (ACR 30–300 mg/g, previously called microalbuminuria); severely increased albuminuria (ACR > 300 mg/g, previously called macroalbuminuria).

### Statistical analyses

PASW Statistics 18.0 statistical software (SPSS Inc., Chicago, IL, USA) and Microsoft Office Excel 2003 were used for data analysis. The expression of eGFR equation in PASW: eGFR = EXP(LN(151) − 0.328 * LN(Scr/88.4/0.7) + age * LN(0.993)) (If female and creatinine <0.7); =EXP(LN(151) − 1.210 * LN(Scr88.4/0.7) + age * LN(0.993)) (if female and creatinine >=0.7); =EXP(LN(149) − 0.412 * LN(Scr/88.4/0.9) + age * LN(0.993)) (If male and creatinine <0.9); =EXP(LN(149) − 1.210 * LN(Scr/88.4/0.9) + age * LN(0.993)) (If male and creatinine >=0.9).

Data were collected by mean ± SD. Prevalence of different thyroid diseases among different ACR groups and CKD categories were analyzed by chi-square test. Levels of thyroid hormone were compared among different ACR groups by Kruskal Wallis test. We calculated the prevalence of different thyroid diseases among three ACR groups. Association of albuminuria and thyroid hormone was explored via correlation analysis. *P* values < 0.05 were considered to be statistically significant.

## Results

### Characteristics of subjects in different ACR groups

**Figure 1:**
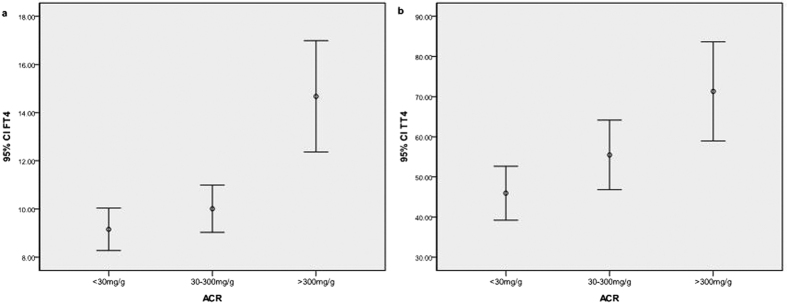
The mean and 95% CI of FT4 and TT4 in ACR group. ACR: urinary albumin to creatinine ratio; CI: confidence interval; FT4: free T4 (pmol/L); TT4: total T4 (mmol/L).

**Figure 2:**
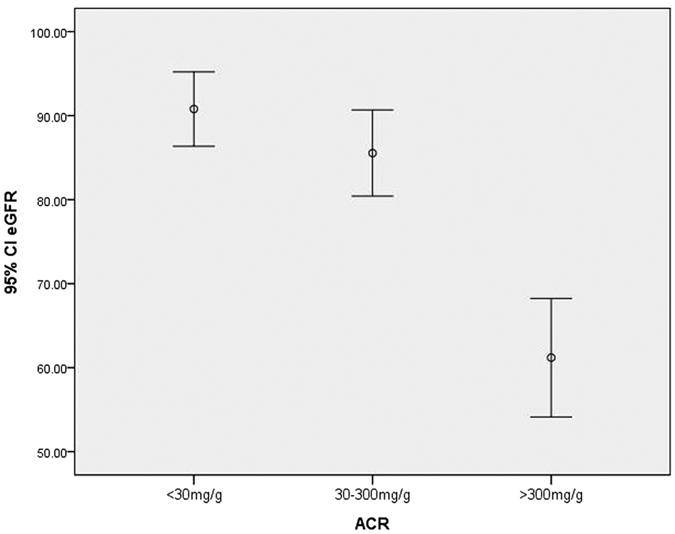
The mean and 95% CI of FT4 and TT4 in CKD stage. CKD stage 1: eGFR ≥ 90 ml/min; CKD stage 2: 90 ml/min > eGFR ≥ 60 ml/min; CKD stage 3: 30 ml/min > eGFR ≥ 60 ml/min; CKD stage 4: 15 ml/min > eGFR ≥ 30 ml/min; CKD stage 5: eGFR < 15 ml/min; CI: confidence interval; FT4: free T4 (pmol/L); TT4: total T4 (mmol/L).

A total of 581 subjects with CKD were enrolled. The causes of CKD cases were chronic glomerulonephritis, asymptomatic hematuria, diabetic nephropathy, polycystic kidney disease, chronic renal failure (unknown or indeterminate aetiology), hypertensive nephropathy, and obstructive nephropathy. There was a significant difference among the three groups in FT4, TT4 (Fig. 1), Scr and eGFR. eGFR in ACR > 300 mg/g group was significantly lower than other groups (*P* < 0.001) (Fig. 2). There was a positive correlation between FT4, TT4 with ACR. FT4 or TT4 was increased according to the incremental ACR with those of ACR > 300 mg/g being the highest. No significant difference was found among groups in FT3 (*P* = 0.790), TT3 (*P* = 0.296), TSH (*P* = 0.746), sex (*P* = 0.374) and age (*P* = 0.735) by Kruskal Wallis test (Table 2).

### The prevalence of different thyroidism in each ACR group

Euthyroid sick syndrome and hypothyroidism (subclinical and clinical) were more common in CKD patients. There was a significant difference in the prevalence of different thyroidism among three ACR groups (*P* = 0.029). Euthyroid sick syndrome and hypothyroidism were both more prevalent than hyperthyroidism in each ACR group. There was an increasing trend for the prevalence of euthyroid sick syndrome with the decrease of the ACR (Table 3).

No significant association of ACR with different clinical categories of thyroid dysfunction was found by Spearman analysis (*P* = 0.864).

### The prevalence of different thyroidism in each CKD stage

There was a significant difference in the prevalence of different thyroidism among 5 groups of CKD stage (*P* = 0.004). The prevalence of euthyroid sick syndrome in CKD stage 4 and stage 5 group was higher than other groups. There was an increasing trend for the prevalence of euthyroid sick syndrome with the decrease of the eGFR, showed a U-curve (Table 4).

### Thyroid hormone correlation with albuminuria

**Figure 3:**
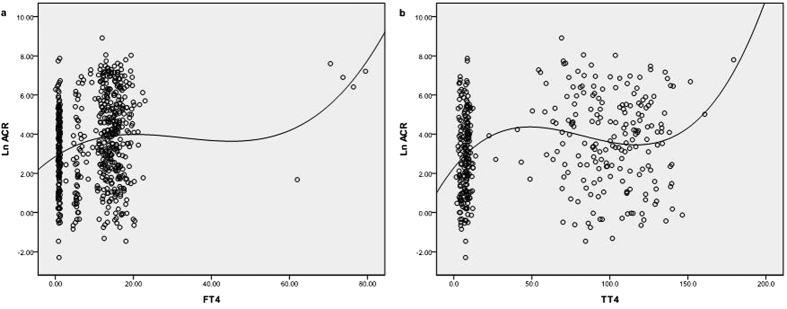
The scatterplot of FT4 and TT4 associated with Ln ACR. Smoothed line shows the fit of the dat. FT4: free T4 (pmol/L); TT4: total T4 (mmol/L); Ln ACR: urinary albumin to creatinine ratio by natural logarithm transformed.

The scatterplot of TT4 associated with ACR (95% CI) was shown in Fig. 3a. Spearman correlation coefficient (r) was 0.165, *P* = 0.001, indicating that TT4 was significantly positively correlated with ACR.

The scatterplot of FT4 associated with ACR (95% CI) was shown in Fig. 3b. Spearman correlation coefficient (r) was 0.162, *P* < 0.001, indicating that FT4 was significantly positively correlated with ACR.

TT3, FT3 and TSH were not associated with ACR (*P* = 0.704, 0.515 and 0.951, respectively).

Albuminuria was an independent variable of FT4 after reduced adjustment for age, sex, serum albumin, hs-CRP, smoking status, systolic blood pressure, diabetes mellitus, medication use for diabetes mellitus (*P* < 0.001) and full adjustment for all of the above mentioned plus eGFR, LDL cholesterol, triglycerides, hypertension, medication use for hypercholesterinemia (*P* = 0.006).

Albuminuria was an independent variable of TT4 after reduced adjustment for age, sex, serum albumin, hs-CRP, smoking status, systolic blood pressure, diabetes mellitus, medication use for diabetes mellitus (*P* = 0.013) and full adjustment for all of the above mentioned plus eGFR, LDL cholesterol, triglycerides, hypertension, medication use for hypercholesterinemia (*P* = 0.039).

We did not show significant association of the presence of thyroid disease with albuminuria (*P* = 0.989) by bivariate Logistic regression analysis.

## Discussion

In our study, we demonstrated that TT4 and FT4 were significantly different in three ACR groups that classified as <30 mg/g, 30–300 mg/g and >300 mg/g (both *P* < 0.001). Positive correlation between TT4, FT4 and albuminuria was evaluated by correlation analysis (Spearman correlation coefficient were 0.162 and 0.165, respectively, with *P* = 0.001 and <0.001). Albuminuria was an independent variable of T4 (TT4 and FT4) after reduced adjustment and full adjustment. In contrast, TT3, FT3 and TSH were not associated with albuminuria. Moreover, we showed that there was a significant difference in the prevalence of different thyroidism among three ACR groups (*P* = 0.029). Euthyroid sick syndrome and hypothyroidism were more prevalent than hyperthyroidism in CKD. The prevalence of euthyroid sick syndrome was 38.3% in normal albuminuria patient (defined as ACR < 30 mg/g).

Thyroid hormone affects nearly every organ system in the body. T4 is produced only by the thyroid gland, whereas T3, the more biologically active form of thyroid hormone, is produced primarily through local deiodination of T4 by the enzyme T4–5′-deiodinase in other tissues, including the kidney. The kidney contains the D1 isoform of this enzyme, which becomes less active in CKD^18^. The kidney plays a role in clearance of iodine, TSH, and thyrotropin-releasing hormone. However, some patients with CKD are euthyroid, with normal TSH and free T4 levels. Patients with CKD may have changes in thyroid function tests consistent with the euthyroid sick syndrome; that is, low T4, T3, and TSH concentrations. End stage renal disease (ESRD) patients have decreased levels of free T3. These changes in CKD patients are due to alterations in the peripheral 5′-monodeiodination of T4, reduced levels of plasma proteins that bind T4, the presence of inhibitors of T4 binding to plasma proteins, metabolic acidosis, and effects of medications^18^. Patients with nephrotic syndromes have urinary losses of proteins that bind thyroid hormones, including thyroxine binding globulin, transthyretin, and albumin. Urinary T4 excretion was measured in patients with proteinuria. One study showed that, it was detectable in the urine in five cases, who had significantly lower serum free T4 and free T3 concentrations than the five patients without detectable urinary T4^11^. This can result in reductions in total plasma T4 and less commonly total T3 levels that are roughly proportional to the severity of hypoalbuminemia and degree of proteinuria. Many such patients remain euthyroid, however, as the result of increased secretion of TSH and thyroid hormone synthesis, albeit clinical hypothyroidism can occur. Heparin and furosemide can inhibit T4 binding to plasma proteins and may transiently elevate free T4 levels^18^. No previous study focused on the association between thyroid dysfunction and albuminuria. Our study showed that TT4 and FT4 were both higher in macroalbuminuria, which were completely not consistent with our anticipation. There might be several possible reasons for the higher TT4 and FT4 in macroalbuminuria. Firstly, the higher T4 is probably related to glomerular hyperfiltration and hypertension, changes in tubular protein handling, or changes in the structure of glomerular barrier, all of which may increase albuminuria^19^. The higher T4 (or hyperthyroidism) may result in glomerular hyperfiltration^20^. Secondly, thyroid gland is able to compensate for hormonal urinary losses keeping the patient euthyroid in macroalbuminuria^10^. Although TT4 and FT4 were both higher in macroalbuminuria group than microalbuminuria group and nomal albuminuria group, serum TT4 and FT4 did not exceed reference ranges in most patients in our study.

Hypothyroid humans and rats can have an increased transcapillar leaking of the plasma proteins such as albumin, which leads to mild proteinuria and albuminuria^19^. The albuminuria is considered to be present before the decrease in GFR in hypothyroid patients^19^, which proved our study by showing that 17.9% of microalbuminuria patients and 22.4% of macroalbuminuria patients had hypothyroidism. Patients with proteinuria have higher TSH levels, consistent with urinary loss of thyroid hormones^12^. A possible association between subclinical hypothyroidism and albuminuria were examined in 159 people with type 2 diabetes^21^. Patients with subclinical hypothyroidism had significantly higher levels of ACR than those with euthyroidism. Multivariate logistic regression analyses demonstrated that serum TSH level was an independent risk factor of albuminuria in this study^21^. However, our study showed that albuminuria was not associated with TSH, attributed to most patients without type 2 diabetes in our study.

Subclinical hypothyroidism and euthyroid sick syndrome are common in chronic kidney disease^22^,^23^,^24^. A study showed that low T3 syndrome was highly prevalent in CKD and was a remarkable finding in early CKD^25^. An about 60% prevalence of euthyroid sick syndrome (nonthyroidal illness syndrome) was revealed in aged hospitalized patients^13^. These findings were similar to our results. Our study showed that more than half patients had lower TT4 and/or FT4 across a wide range of ACR (Fig. 1), which resulted in highly prevalence of euthyroid sick syndrome, especially at normal ACR.

Low T3 is the most frequent alteration of the thyroid hormone profile observed in CKD^26^. This alteration has long been considered an innocent metabolic adaptation to chronic illness. However, low T3 associates with endothelial dysfunction, a harbinger of atherosclerosis, in stage 3 and 4 CKD patients, as well as with cardiovascular disease^27^, mortality and with a high risk of death in stage 5D CKD patients^28^.

The strength of this study is the power. To detect a small-moderate correlation (for example r = 0.13), a sample of 500 analyzable subjects will provide 83% power to discover that the correlation is significantly different from there being no correlation (i.e. that the correlation would be zero) at the 0.05 level. The correlation coefficient is more than 0.13 and the sample size of analyzable subjects is more than 500 in this study, and therefore, the power of this study is very strong.

There are some limitations to this study. Firstly, we did not test urinary total protein in our research. Therefore, the association of proteinuria and thyroid dysfunction was not performed. However, the association of proteinuria and thyroid dysfunction was well studied^7^,^8^,^9^,^10^,^11^,^12^. Secondly, the data is based on Chinese patients with CKD, thus, it is not clear whether it is applicable to other racial population. Thirdly, as it is a cross-sectional study, the cause and effect cannot be determined from this data. A selection or systematic bias or other confounding factors may also occur. Fourthly, low case numbers for the different clinical categories of thyroid dysfunction in CKD patients is another limitation, for example, only 14 CKD patients with hyperthyroidism were included in this study.

In conclusion, our results showed that serum TT4 and FT4 were positively correlated with albuminuria, and it was completely not consistent with our anticipation. Albuminuria was not associated with TT3, FT3 and TSH. Further study is needed to elucidate the causal association between albuminuria and T4.

## Additional Information

**How to cite this article:** Du, X. *et al*. Albuminuria is an independent risk factor of T4 elevation in chronic kidney disease. *Sci. Rep.* **7**, 41302; doi: 10.1038/srep41302 (2017).

**Publisher's note:** Springer Nature remains neutral with regard to jurisdictional claims in published maps and institutional affiliations.

## Figures and Tables

**Table 1:** The CKD-EPI four-level race GFR estimation equation (Asian people).

Sex	Serum Creatinine Level, μmol/L (mg/dL)	Equation
Female	≤62 (≤0.7) > 62 (>0.7)	151 × (0.993)^Age^ × (Scr/0.7)^−0.328^151 × (0.993)^Age^ × (Scr/0.7)^−1.210^
Male	≤80 (≤0.9) > 80 (>0.9)	149 × (0.993)^Age^ × (Scr/0.9)^−0.412^149 × (0.993)^Age^ × (Scr/0.9)^−1.210^

**Table 2:** Characteristcs of subjects in different ACR group.

Characteristics	ACR < 30 mg/g	ACR 30–300 mg/g	ACR > 300 mg/g
FT3 (pmol/L)	3.54 ± 1.99	3.56 ± 1.10	3.57 ± 1.01
TT3 (mmol/L)	1.17 ± 0.58	1.18 ± 0.39	1.23 ± 0.59
FT4 (pmol/L)**	9.15 ± 12.44	10.01 ± 6.96	14.68 ± 12.58
TT4 (mmol/L)*	45.90 ± 48.50	55.46 ± 50.71	71.32 ± 49.03
TSH (mIU/L)	4.68 ± 6.05	5.63 ± 21.80	4.62 ± 10.03
ACR (mg/g)	8.89 ± 8.14	112.15 ± 69.90	996.15 ± 843.73
Age	63.69 ± 15.69	62.56 ± 15.73	63.72 ± 15.54
Sex (male/female)	105/164	89/107	50/56
Scr (mg/dL)**	1.05 ± 0.80	1.23 ± 1.10	1.87 ± 1.29
eGFR (ml/min)**	90.79 ± 36.87	85.54 ± 36.40	61.19 ± 38.38

ACR: urinary albumin to creatinine ratio; Scr: serum creatinine; eGFR: estimated glomerular filtration rate by CKD-EPI four-level race equation (*P = 0.007; **P < 0.001 by Kruskal Wallis test).

**Table 3:** The prevalence of different thyroidism in each ACR group.

Group	Normal TH	Hyperthyroidism	Hypothyroidism	Euthyroid Sick Syndrome
ACR1 N = 269	97 (36.1%)	2 (0.7%)	67 (24.9%)	103 (38.3%)
ACR2 N = 196	66 (33.7%)	10 (5.1%)	35 (17.8%)	85 (43.4%)
ACR3 N = 116	47 (40.5%)	2 (1.7%)	26 (22.4%)	41 (35.4%)

Clinical and subclinical categories of hypo-/hyperthyroidism were collapsed. ACR 1–3: urinary albumin to creatinine ratio < 30 mg/g, 30–300 mg/g and >300 mg/g, respectively; TH: thyroid hormone (χ^2^ = 14.085, *P* = 0.029 by chi-square test).

**Table 4:** The prevalence of different thyroidism in each CKD stage.

CKD stages N = 581	Normal TH N = 210 (36.2%)	Hyperthyroidism N = 14 (2.4%)	Hypothyroidism N = 128 (22%)	Euthyroid Sick Syndrome N = 229 (39.4%)
1N = 287	101 (35.2%)	7 (2.4%)	59 (46.1%)	120 (41.8%)
2N = 122	45 (36.9%)	3 (2.5%)	27 (22.1%)	47 (38.5%)
3N = 108	46 (42.6%)	1 (0.9%)	28 (25.9%)	33 (30.6%)
4N = 46	12 (26.1%)	2 (4.3%)	12 (26.1%)	20 (43.5%)
5N = 18	6 (33.3%)	1 (5.6%)	2 (11.1%)	9 (50.0%)

Clinical and subclinical categories of hypo-/hyperthyroidism were collapsed. CKD stage 1: eGFR ≥ 90 ml/min; CKD stage 2: 90 ml/min > eGFR ≥ 60 ml/min; CKD stage 3: 30 ml/min > eGFR ≥ 60 ml/min; CKD stage 4: 15 ml/min > eGFR ≥ 30 ml/min; CKD stage 5: eGFR < 15 ml/min; TH: thyroid hormone (χ^2^ = 34.89, *P* = 0.004 by chi-square test).
